# 

**Published:** 1871-12

**Authors:** 


☞ Should any of our subscribers or exchanges fail to get The Companion regularly, they will confer a favor by notifying us of the fact.

☞ We respectfully solicit Original Articles, Reports of Societies, Clinics Home and Foreign Correspondence, News, etc., etc., of interest to medical readers.

☞ To insure publication, articles should be practical, as brief as possible, and carefully written, on one side of the paper. Articles should be received by the 15th of one month to insure publication in the next. ☞ Friends, send in your articles. ☜

---

### OUR THANKS,

### BOOKS AND PAMPHLETS.

*The Druggist's General Receipt-Book* :—Comprising a copious Veterinary Formulary; with numerous recipes in patent and proprietary medicines, Druggist's nostrums, etc. Perfumery and Cosmetics, Beverages, Deletic Articles, and Condiments, Trade Chemicals, Scientific processes, and an Appendix of useful tables, by Henry Beasley, author of "Book of Prescriptions," etc., etc. Seventh American Edition, from the last London Edition. Philadelphia, Lindsay & Blakiston, 1871.

We have been favored by the publishers, with a copy of the above work. After a careful examination, we cordially recommend it. The seventh edition is an improvement on former editions, and will be found to embrace many useful facts for the physician. No Druggist should be without it; and the country physician, who must be his own apothecary as well, will find it an excellent friend and adviser on many occasions.

*The Physician's Dose and Symptom Book*—Containing the dose and uses of all the principal articles of the Materia Medica and officianal preparations; also, Table of weights and measures; rules to proportion the doses of medicines; common abreviations, etc., etc.; table of poisons and antidotes; Pharmaceutical preperations; table of Symptomatology; outlines of general Pathology and Therapeutics, by Joseph H. Wythes, A. M. M. D. Tenth Edition, Philadelphia, Lindsay & Blakiston, 1871.

As a general rule, we are opposed to "*Vade Mecum's*" which too often seek to furnish lazy students a passable quantity of information at the expense of little study and labor. Such men dishonor medicine and bring a reproach upon science. If, however, some men will not study, and medical colleges, for the perquisites, will graduate them, then, for the sake of humanity, let them have a good Vade Mecum, such as Dr. Whythe's. It will, at least learn them many things they would never acquire without it.

To the Student—the real man of Science—a scientific Vade Mecum is of value as a "remembrancer." For this purpose, and as a convenience to the busy practitioner, we can heartily recommend the useful and valuable little "pocket dose book."

*The Physicians' Visiting List, for* 1872.—We are indebted to the publishers, Lindsay & Blakiston, No. 22, S. Sixth street, Philadelphia, for a copy of the above. This visiting list is intended for 25 patients per week. It is compact and answers the intended purpose admirably.

*Modern Medical Therapeutics.*—A compendium of recent formula and special therapeutical directions, by Geo. H. Napheys, A. M., M. D. Third Edition, revised and improved, Philadelphia, S. W. Butler, M. D., 115 South Seventh street, 1871.

We have been favored with the above work by the publisher, Dr S. W. Butler, editor and proprietor of that excellent Medical Weekly, the Medical and Surgical Reporter. We have before noticed the Second Edition of Dr. Napheys' valuable work. The formulæ are elegant, and from the most distinguished medical men of Europe and this country. The value of these formulæ is very greatly enhanced by the "specific therapeutical directions" and the judicious classification and arrangement of the diseases for which they are employed. The Third Edition has been increased by seventy pages of useful and interesting matter. The practitioner will find in this volume a fund of valuable information, hints and suggestions, which cannot fail to prove of great advantage.

*Cancer—Its Classification and Remedies.*—By J. W. Bright, M. D., Philadelphia. Published by S. W. Butler, M. D., 1871.

Dr. Bright has given the profession a work which will add very materially to the literature upon that opprobium of medicine and surgery—Cancer. The author has done his work well, his aim having been "to place in the hands of the profession a work that will save them much labor and toil in collecting facts, and at the same time a classification that will remove the difficulty of having so many names for the same form of Cancer."

The work is readable, exceedingly interesting, and will prove, without doubt, a valuable contribution upon a disease not sufficiently well understood by the profession at large. Like the work of Dr. Napheys, the typographical execution is excellent, and creditable to the worthy and energetic publisher, Dr. Butler.

*The Physician's Daily Pocket Record*—Comprising a visiting list, many useful memoranda, tables, etc., by S. W. Butler, M. D., Philadelphia. We have used the Daily Pocket Record of Dr. Butler, and have found it one of the best published.

*A Report of Surgical Cases Treated in the Army of the United States from* 1865 *to* 1871.

We are under obligation and tender our thanks to the Surgeon General, Joseph K. Barnes, M. D., of the United States Army, for "Circular No. 3," with title as above, and prepared by Geo. A. Otis, M. D., Assistant Surgeon, U. S. A. The volume is exceedingly interesting, being finely illustrated, and will prove a valuable acquisition to the Surgical literature of the times. We regret that want of space must abridge our notice of the merits of this instructive and valuable report. We hope, however, to allude, specially, to a few of the many interesting cases given, at some future time.

*Medical Education in Amcrica*—Being the Annual Address read before the Massachusetts Medical Society, June 7th, 1871, by Henry J. Bigelow, M. D., Professor of Surgery in Harvard University, Cambridge. Welch, Bigelow & Co., 1871.

We have read the address of Prof. Bigelow, with very great satisfaction and pleasure. There are some things in it which we ought not and cannot endorse, but in the main, it is a truthful and able expose of the present status of Medical Education in America. As one of the representatives of Harvard University, he can speak boldly and strongly in defence of a more extensive and thorough medical preparation on the part of the student, both before and after matriculation. We would be recreant to our feelings and unjust to the profession we desire to see honored and elevated, not to express our hearty and sincere approval of the recent effort made by Harvard University to improve medical education. We hope, very soon, to see this wise and honorable step followed by every other Medical Institution in the country.

*Clinical Examination of the Urine*—With a description of a convenient apparatus for its speedy analysis. By Reuben A. Vance, M. D. Wm. Baldwin & Co., New York.

Those of our readers who desire to become more expert in the examination of urine, will do well to procure Dr. Vance's little pamphlet.

*Transactions of the Mississippi State Medical Society*—Held at Meridian, April 3d and 4th, 1871.

We return our thanks to the Recording Secretary, Dr. J. W. M. Shattuck, for the above. Our associate, Dr. S. V. D. Hill was the retiring President. The proceedings give evidence of great professional activity, both in maintaining the dignity of medicine and in promoting the cause of science. We wish our brethren God speed in their noble work. We regret our want of space denies us the pleasure of noticing the proceedings at greater length.

*Syphilictic Epilepsy.*—By Reuben A. Vance, M. D.
This pamphlet will be found of practical importance to every practitioner.

*The Prevention of Abscesses in Hypodermic Medication*—With a description of an instrument for the injection of strychnia. By Reuben A. Vance, M. D. Wm. Baldwin & Co, 12 Parke Row, New York, 1871.

We have before noticed Dr. Vance's pamphlet on the use of Strychnia hypodermically, in paralysis. The above will give the practitioner some very useful hints in the use of the hypodermic syringe.

*A Contribution* to the treatment of the Versions and Flexions of the unimpregnated Uterus, by Ephraim Cutter, A. M., M. D.,

Boston. James Campbell, publisher, 18 Tremont street, 1871. Price, 50 cents.

We are indebted to the publisher for the excellent mongraph of Dr. Cutter. The subjects are well handled. The principles of treatment for retroversion laid down by the author, are as follows:

1st. To restore the uterus and vagina to their normal place by the uterine sound.

2d. To maintain them there by means—mechanically adapted to the parts—which allow of the normal contraction of the transverse vaginal fibers, and which permit a natural degree of uterine imobility.

3d. To have unirritative material.

4th. To be manageable by the patient.

5th. To pay attention to the general health.

The author endeavors to carry out these principles, by two forms of pesseries—the *loop* and the *T.* Wood-cuts are given by which the application and adoption of those pesseries to the various parts are demonstrated. That they will prove efficient means of relief to sufferers of retroversion, we hope and believe.

The pamphlet is certainly the most complete and exhaustive monograph upon the subjects proposed, which we have seen, and justice requires us to say they are most admirably and satisfactorily discussed by the author. The pamphlet should be in the hands of every physician who desires to be well informed upon the subjects of which it treats.

*Report of Six Hundred Cases of Diseases of the Ear*—By Lawrence Turnbull, M. D., Physician to the Department of Diseases of the Eye and Ear in the Howard Hospital, Philadelphia.

The above named pamphlet very succinctly gives a number of diseases of the eye and ear, with the treatment, when necessary. It will prove serviceable to not only those desiring to study, specially, the diseases of the eye and ear, but the general practitioner as well.

